# Room Temperature Uniaxial Magnetic Anisotropy Induced By Fe‐Islands in the InSe Semiconductor Van Der Waals Crystal

**DOI:** 10.1002/advs.201800257

**Published:** 2018-05-11

**Authors:** Fabrizio Moro, Mahabub A. Bhuiyan, Zakhar R. Kudrynskyi, Robert Puttock, Olga Kazakova, Oleg Makarovsky, Michael W. Fay, Christopher Parmenter, Zakhar D. Kovalyuk, Alistar J. Fielding, Michal Kern, Joris van Slageren, Amalia Patanè

**Affiliations:** ^1^ School of Physics and Astronomy The University of Nottingham NG7 2RD Nottingham UK; ^2^ Department of Physics Chemistry and Biology Linköping University 581 83 Linköping Sweden; ^3^ National Physical Laboratory Hampton Road TW11 0LW Teddington UK; ^4^ Nanoscale and Microscale Research Centre The University of Nottingham NG7 2RD Nottingham UK; ^5^ Institute for Problems of Materials Science The National Academy of Sciences of Ukraine 58001 Chernivtsi Ukraine; ^6^ School of Pharmacy and Biomolecular Sciences Byrom Street L3 3AF Liverpool UK; ^7^ Institut für Physikalische Chemie and the Center for Integrated Quantum Science and Technology Universität Stuttgart Pfaffenwaldring 55 70569 Stuttgart Germany

**Keywords:** electron spin resonance (ESR), InSe, iron, magnetic anisotropy, van der Waals semiconductors

## Abstract

The controlled manipulation of the spin and charge of electrons in a semiconductor has the potential to create new routes to digital electronics beyond Moore's law, spintronics, and quantum detection and imaging for sensing applications. These technologies require a shift from traditional semiconducting and magnetic nanostructured materials. Here, a new material system is reported, which comprises the InSe semiconductor van der Waals crystal that embeds ferromagnetic Fe‐islands. In contrast to many traditional semiconductors, the electronic properties of InSe are largely preserved after the incorporation of Fe. Also, this system exhibits ferromagnetic resonances and a large uniaxial magnetic anisotropy at room temperature, offering opportunities for the development of functional devices that integrate magnetic and semiconducting properties within the same material system.

Magnetic anisotropy is at the heart of spintronics: it gives rise to an energy barrier between two opposite spin directions and hence to the possibility to store and process information.^1^ Magnetic anisotropy has been demonstrated in several material systems, ranging from magnetic ion nanoclusters^2^ and single‐molecule magnets^3^ to single atoms.^4^ However, the integration of magnetic and semiconducting properties within the same material system is generally difficult to achieve. This integration is important for several applications and new device concepts in spintronics, including electrical control of the magnetization and generation of spin‐currents,^5^ spin‐filtering^6^ and spin‐amplification^7^ in logic devices. To date, the realization of magnetic semiconductors has proven to be challenging and it has led to material systems with interesting magnetic and electronic properties.^8^, ^9^, ^10^

In this work, we demonstrate magnetic and semiconducting properties in the van der Waals (vdW)‐layered crystal InSe, a material system that has emerged as a promising candidate for electronics^11^ and photonics^12^ due to its high electron mobility, chemical stability, and high photoresponsivity. In a vdW crystal, the atoms in each layer are bound by strong covalent bonds, whereas the planes are held together by weak vdW interactions. The extended family of vdW crystals includes graphene, hexagonal boron nitride, transition metal dichalcogenides, and many others. Although a variety of semiconductor crystals and stacks has been demonstrated, the available structures are nonmagnetic, weakly magnetic, or magnetic only at low temperature.^13^ Here, we show that InSe, which is nonmagnetic in its pristine form, becomes magnetic following the incorporation of Fe‐atoms during the growth of InSe by the Bridgman method. We show that the Fe‐atoms self‐assemble into islands embedded within the InSe host‐crystal. The islands are crystalline and have a triangular shape in the plane of the vdW layers. Our material tends to retain the electronic, optical, and vibrational properties of pristine InSe. However, the Fe‐islands imprint the InSe crystal with a large uniaxial magnetic anisotropy at room temperature with the magnetization preferentially oriented in the direction perpendicular to the plane of the vdW layers. Thus, room temperature magnetism and semiconducting properties are achieved within the same material system, offering opportunities for further research developments and exploitation.

The γ‐polytype InSe and the Fe‐doped γ‐InSe crystals were grown using the Bridgman method from a polycrystalline melt of In_1.03_Se_0.97_. Fe‐dopants were incorporated during the growth at a nominal concentration of 1% and 10% (Experimental Section). The primitive unit cell of γ‐InSe contains three layers each of which has a thickness of *L* = 8.320 Å and consists of four covalently bonded monoatomic sheets in the sequence Se‐In‐In‐Se; along the *c*‐axis, the primitive unit cell has a lattice constant *c* = 24.961 Å; within each *a–b* plane atoms form hexagons with lattice parameter *a* = 4.002 Å (**Figure** **1**a). The lattice parameters are weakly modified following the incorporation of Fe, as probed by X‐ray diffraction (Experimental Section and Section S1, Supporting Information). However, studies of the crystals by spatially resolved energy‐dispersive X‐ray (EDX) spectroscopy and electron diffraction reveal that the Fe‐atoms self‐assemble into crystalline islands that are randomly oriented in the *ab*‐plane (Figure 1b). In the thicker films (>1 µm), these islands are elongated along the *c*‐axis (Figure 1c). These nanostructures contain a high content of Fe (>95%) and have a triangular shape in the *ab*‐plane, as shown in the high‐resolution EDX image of **Figure** **2**a. In this figure, the two equal sides of the triangular Fe‐islands have length of 0.8 µm. Furthermore, the Fe‐atoms are arranged into a body centered cubic (bcc) lattice with lattice constant *a* = 2.87 Å (Figure 2b), as for bulk γ‐Fe. Thus, Fe‐islands with a bcc lattice (Figure 2b) coexist with the rhombohedral crystal structure of γ‐InSe (Figure 2c). The low solubility of Fe in InSe and the large Fe‐content create supersaturation conditions during the Bridgman growth of γ‐InSe, thus leading to the formation of two distinct crystals within the same material system (Experimental Section).

**Figure 1:**
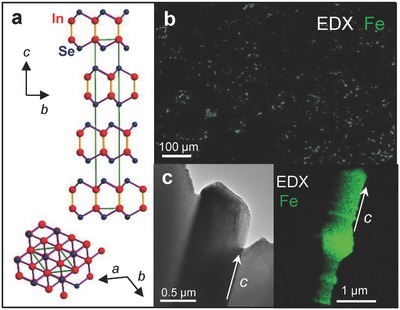
Fe‐islands in InSe van der Waals crystals. a) Schematic representation of the crystal structure of InSe. b) SEM‐EDX maps of the surface of an InSe crystal grown by the Bridgman method with a nominal Fe‐content of 10%. c) Cross‐sectional TEM image and EDX map of an Fe‐island in InSe. The island is elongated along the *c*‐axis.

**Figure 2:**
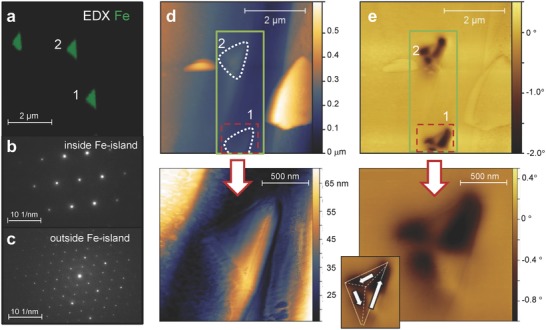
Triangular‐shaped Fe‐islands and magnetic domains. a) TEM‐EDX in‐plane map of an InSe thin film (thickness of 100 nm) with nominal Fe content of 10%. Two specific Fe‐islands are labelled as 1 and 2. b,c) Electron diffraction patterns b) within and c) outside one of the Fe‐islands shown in part (a). The two diffraction patterns correspond to a bcc Fe‐lattice and a rhombohedral InSe crystal, respectively. d) Top: AFM image of the InSe film shown in part (a), with Fe‐islands outlined in white. The region corresponding to the Fe‐islands #1 and #2 is marked by a green rectangle. Bottom: enlarged AFM image of Fe‐island #1. e) Top: MFM image showing the multidomain structure of Fe‐islands #1 and #2 (*h* = 25 nm). Bottom: enlarged MFM image for Fe‐island #1 (*h* = 50 nm). Inset: sketch of the multidomain structure.

Magnetic force microscopy (MFM) measurements were conducted in the thermally demagnetized state of the crystals using a two‐pass method and a CoCr‐tip magnetized along the tip axis. The first pass was conducted in a tapping mode to reveal the surface topography (e.g., atomic force microscopy, AFM); this was then followed by the second pass at an increased scan height *h* (25–50 nm) to probe long‐range magnetic interactions. During the MFM imaging, the scan height *h* is maintained constant and the changes in the phase of the oscillating probe are recorded.^14^ These changes originate from the long‐range magnetic interactions between the probe and the sample.

Figure 2d,e shows the AFM and MFM images for a thin InSe film (≈100 nm) at the locations of the Fe‐islands #1 and #2 highlighted in the EDX image of Figure 2a. Dark and bright contrasts in the MFM images (Figure 2e) correspond to magnetic repulsion and attraction, respectively, indicating the presence of perpendicular stray fields emanating from magnetic domains. Both Fe‐islands have a similar domain structure suggesting a close similarity in their magnetic properties (Figure 2e, top). The enlarged AFM and MFM images in the bottom of Figure 2d,e show with greater detail the shape, orientation, and height of an individual Fe‐island and its threefold multidomain state. By comparing the morphology and the domain structure of this island, we conclude that the domain structure is 3D. The largest domain on the right‐hand side morphs around the upper‐right‐hand edge. The two other domains are similar in size, completing the domain closure in an anticlockwise fashion (see schematic representation in the left bottom corner of Figure 2e). These findings demonstrate that ferromagnetic Fe‐islands with a bcc lattice are embedded within the rhombohedral γ‐InSe crystal.

Irrespectively of the Fe‐content, we find that for all our InSe bulk crystals the energy peak position of the room temperature (*T* = 292 K) photoluminescence (PL) emission is centered at an energy *hv* = 1.25 eV (**Figure** **3**a) and the Raman peaks are at 115.7, 179.2, 201.2, 212.4, and 228.0 cm^−1^, as observed for pristine bulk InSe (Figure S2, Supporting Information). With increasing Fe‐content, the intensity of the optical signals tends to decrease and spatial maps of the PL intensity reveal an increasing nonhomogeneity over length scales of a few micrometers (Figure 3b). Correspondingly, the room temperature conductivity in the layer plane decreases due to a reduction of the electron mobility from μ ≈ 10^3^ cm^2^ V^−1^ s^−1^ in pristine InSe to μ ≤ 10^2^ cm^2^ V^−1^ s^−1^ in the InSe crystals containing Fe. Thus, despite the incorporation of Fe‐islands in InSe, the crystal preserves many of the functional properties of pristine InSe. Furthermore, the crystals can be exfoliated into thin layers and the PL emission peak undergoes a strong blueshift Δ*E* with decreasing layer thickness *L* (Figure 3a). The measured energy shift is in agreement with that observed and calculated for pristine InSe (Figure 3a). In our quantum well model, the energy shift is described as Δ*E* = *h* ^2^/8*L* ^2^μ (continuous line in Figure 3a), where *µ* = 0.054 *m*_e_, is the exciton mass and *m*_e_ is the electron mass in vacuum; a similar energy shift is calculated using density functional theory.^15^

**Figure 3:**
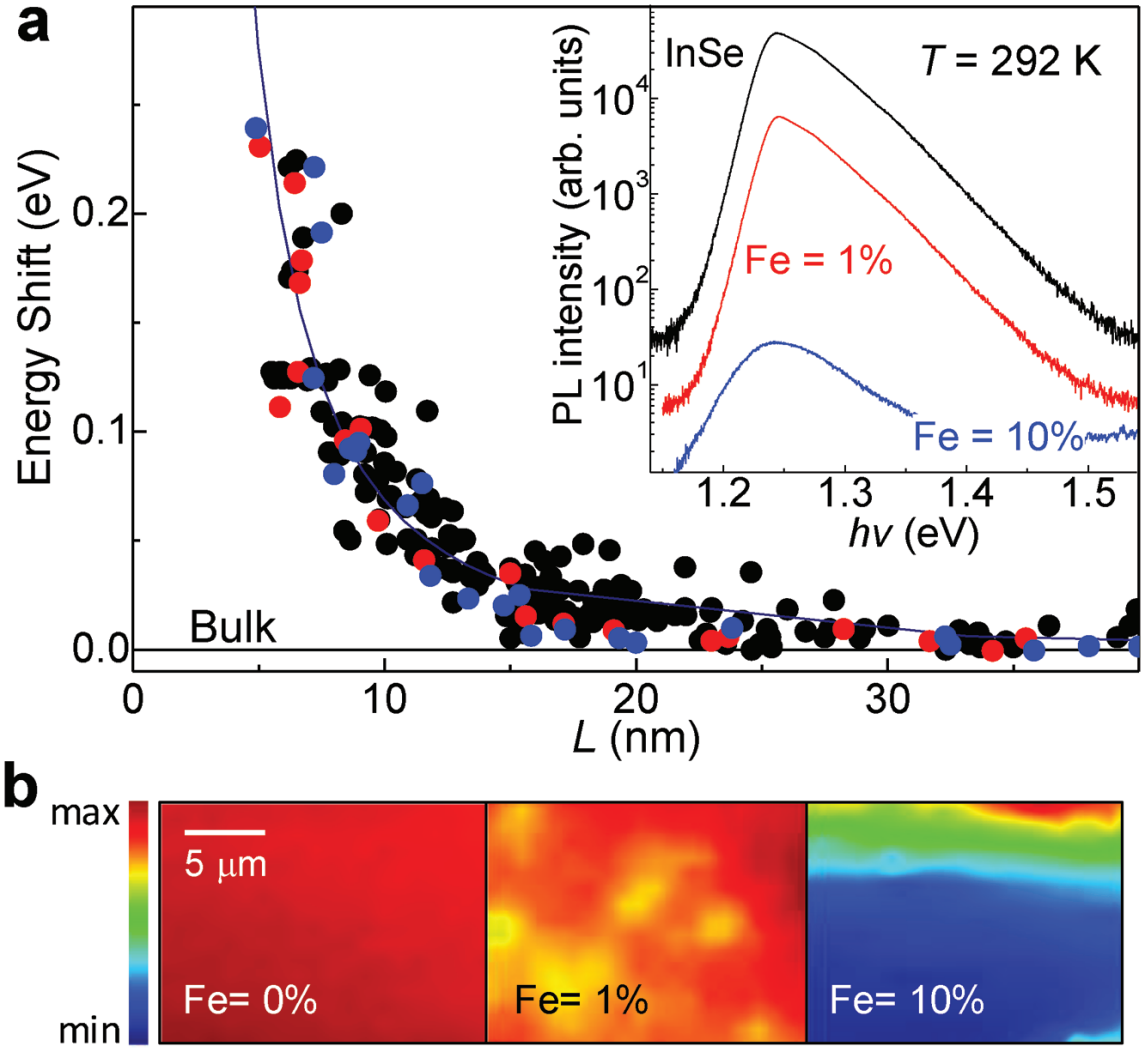
Semiconducting properties of InSe containing Fe‐islands. a) Energy shift of the room temperature PL peak versus the layer thickness *L* for InSe layers with nominal Fe‐content of 0% (black), 1% (red), and 10% (blue). The solid line is the energy shift calculated using an effective mass quantum well model. Inset: PL spectra for bulk InSe samples with and without Fe. b) Maps of the PL intensity for bulk InSe with nominal Fe‐content of 0%, 1%, and 10% (λ = 633 nm; *P* = 0.1 mW).

Although the semiconducting behavior of our samples is preserved, the magnetic properties of InSe are modified after the incorporation of Fe. **Figure** **4**a shows typical room temperature (*T* = 292 K) electron spin resonance (ESR) spectra measured at Q‐band (frequency *v* = 34.229 GHz) for bulk InSe containing Fe‐islands. The experiment is conducted in perpendicular mode, that is, the external magnetic field **B** is perpendicular to the microwave field; also, **B** is at angle *ϑ*_B_ relative to the *c*‐axis (out‐of‐plane geometry, inset of Figure 4a). For **B** parallel to the *c*‐axis (i.e., *ϑ*_B_ = 0°), the ESR spectrum reveals two strong ferromagnetic resonances at *B* = 0.199 and 0.326 T, corresponding to effective *g*‐values of *g*_1_ = 12.3 and *g*_2_ = 7.5, respectively. The position, linewidth, and intensity of the resonances depend on the orientation of **B** with a periodic modulation and turning points that occur when **B** is aligned close to main crystallographic directions, that is, parallel to the *c*‐axis (*ϑ*_B_ = 0°, 180°, and 360°) or to the *ab*‐plane (*ϑ*_B_ = 90° and 270°). For example, the intensity of the main ESR line (*g*_2_) has minima at angles close to *ϑ*_B_ = 90°and 270° (Figure 4b); correspondingly, the resonance field *B*_res_ increases to values of up to ≈1.5 T (Figure 4c) and the resonance linewidth Δ*B* increases by more than a factor of 5 (Figure 4d). This strong magnetic anisotropy is supported by superconducting quantum interference device (SQUID) studies showing a saturation of the magnetization at lower magnetic fields for **B** parallel to the *c*‐axis than for **B** in the *ab*‐plane (Figure S3, Supporting Information). We note that ESR spectra of pristine InSe do not reveal any signal.

**Figure 4:**
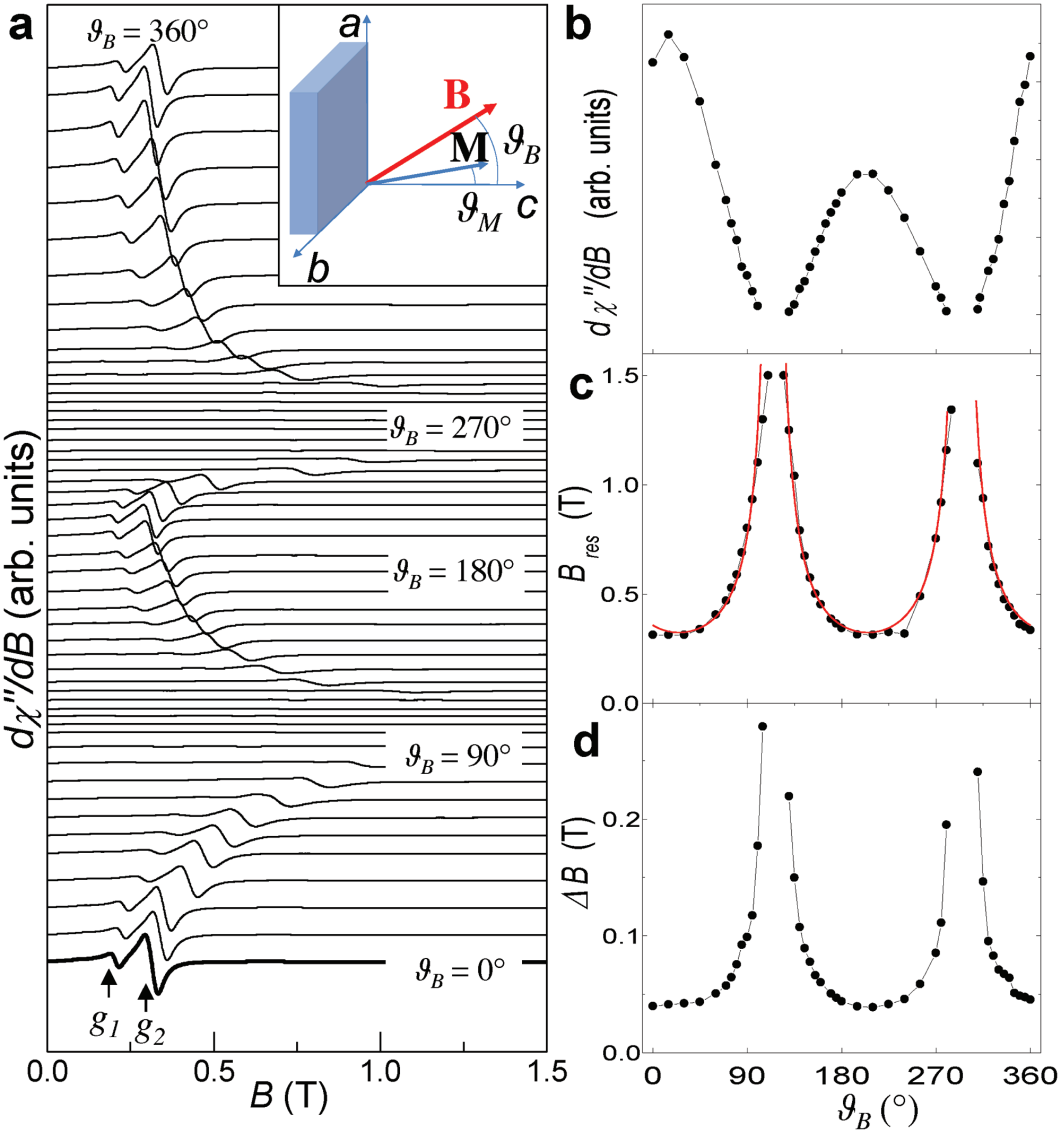
Room temperature uniaxial magnetic anisotropy induced by Fe‐islands in InSe. a) Angle‐dependent ESR spectra of an InSe crystal with nominal Fe content of 10% (Q‐band and *T* = 292K). The ESR spectra are shifted vertically for clarity. The step in the increment of the angle *ϑ*_B_ is 5°. Inset: Orientation of the magnetic field **B** and magnetization **M** relative to the crystallographic *c*‐axis of InSe. b–d) ESR intensity, resonance field *B*_res_, and linewidth Δ*B* of the resonance *g*_2_ versus *ϑ*_B_. The red curve in part (c) is the simulation of the data by Equation (1) in the main text. Black lines in parts (b–d) are guides to the eye.

The ESR resonances are observed for a wide range of temperatures from *T* = 5 to 292 K. **Figure** **5**a shows the *T*‐dependence of the ESR spectra for **B** parallel to the *c*‐axis and the corresponding *T*‐dependent peak‐to‐peak intensity, resonance field, and linewidth for resonance *g*_2_. The ESR intensity decreases steeply from a broad maximum centered at *T* ≈ 260 K to approximately zero for *T* < 50 K (Figure 5b). In the same range of temperatures, *B*_res_ shifts to lower values (Figure 5c) and the ESR linewidth Δ*B* broadens (Figure 5d). Furthermore, for *T* < 100 K, a weak ESR line emerges at *B* = 0.537 T corresponding to *g*_3_ ≈ 4.6 (see also Figure S4 in the Supporting Information). The *T*‐dependent ESR spectra were also acquired for **B** in the *ab*‐plane (Figure S5, Supporting Information). For this orientation of **B**, *g*_1_ and *g*_2_ cannot be clearly resolved; however, a narrow ESR line is observed at *B* = 0.680T (*g*_4_ = 3.6). This has weak dependence on the orientation of **B** in the *ab*‐plane and its *T*‐dependence is similar to that of *g*_1_ and *g*_2_ (Figure S6, Supporting Information).

**Figure 5:**
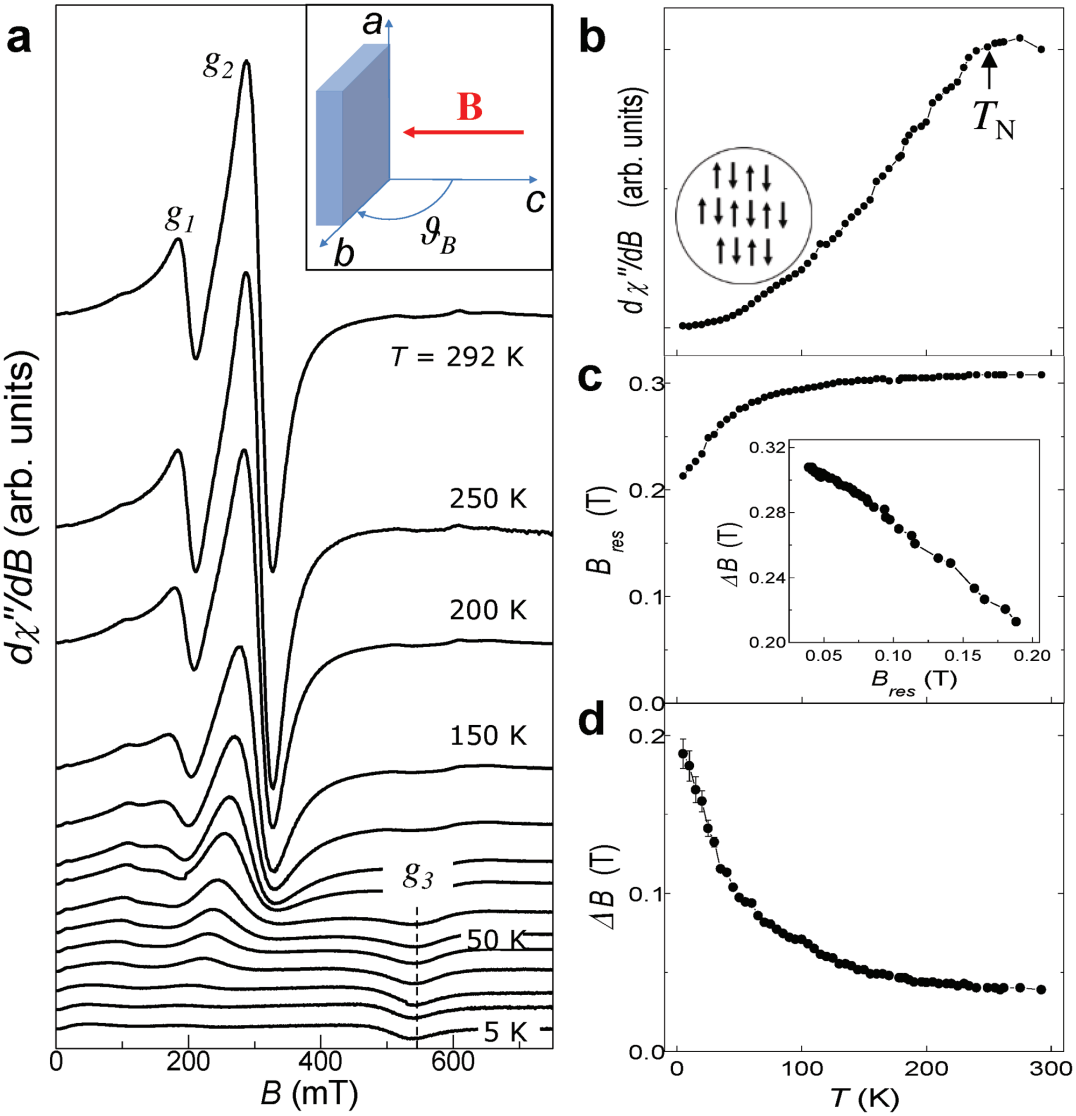
Magnetic phases induced by Fe‐islands in InSe. a) Temperature*‐*dependent ESR spectra for an InSe crystal with nominal Fe = 10% (Q‐band and **B** parallel to the *c*‐axis). Inset: Orientation of **B** relative to the crystallographic *c*‐axis of InSe. The ESR spectra are shifted along the vertical axis for clarity. Temperature values from top to bottom are *T* = 292, 250, 200, 150, 120, 100, 80, 60, 50, 40, 30, 20, 10, and 5 K. b–d) ESR intensity, resonance field *B*_res_ and linewidth Δ*B* of resonance *g*_2_ versus *T*. The inset in part (b) sketches the low *T* antiferromagnetic order. The inset in part (c) shows the correlation between the resonance linewidth and resonance field.

Our crystals combine the electronic properties of the nonmagnetic van der Waals crystal InSe with the magnetic properties of Fe. As shown by MFM (Figure 2e), magnetic domains are observed at room temperature and are localized within the Fe‐islands. Furthermore, the ESR lines correspond to effective *g‐*values (e.g., *g*_1_ = 12.3, *g*_2_ = 7.5, and *g*_3_ = 4.6 for *ϑ*_B_ = 0°), significantly larger than those expected for isolated Fe‐ions, that is, *g* ≈ 4.3 and 2.^16^ The large *g*‐factors and the angular dependence of the ESR lines demonstrate strong ferromagnetic spin–spin interactions in the Fe‐islands, leading to an internal magnetic field with easy axis parallel to the *c*‐axis of InSe.

We model the measured angular dependence of the resonance field *B*_res_ by Equation (1), commonly used for systems with uniaxial magnetic anisotropy in an out‐of‐plane configuration^17^

(1) $$\begin{array}{c} {\left(\frac{\omega }{\gamma }\right)}^{2}=\left[B_{res}\cos\left(\vartheta_{M}-\vartheta_{B}\right)-4\pi M_{eff}\;cos\vartheta_{M}\right]\;\\ \;\;\;\;\;\;\;\;\;\;\;\left[B_{res}\cos\left(\vartheta_{M}-\vartheta_{B}\right)-4\pi M_{eff}\cos^{2}\vartheta_{M}\right] \end{array}$$

Here γ is the gyromagnetic factor, and *ϑ*_M_ and *ϑ*_B_ are the angles between the *c*‐axis and the magnetization, **M**, and external magnetic field, **B**, respectively (inset of Figure 4a). The term 4*πM*_eff_ represents an effective demagnetizing field defined as 4*πM*_eff_ = 4*πM*_s_–2*K*/*M*_s_, where *M*_s_ is the saturation magnetization, *B*_d_ = 4*πM*_s_ is the demagnetizing field, *B*_a_ = 2*K/M*_s_ is the anisotropy magnetic field, and *K* is an anisotropy constant. The simulation of the angular dependence of *B*_res_ in Figure 4c gives *K* = −9 × 10^3^ J m^−3^ and an average anisotropy field ***B_a_*** oriented close to the *c*‐axis with amplitude *B*_a_ ≈ 1 T. We have assumed *M*_s_ = 3 emu g^−1^ (*B*_d_ = 2.7 × 10^−1^ T), as obtained from our SQUID studies (Figure S3, Supporting Information), and γ = 2π*gµ*_B_/*h* with *g* = 2.09.^18^

We describe the angular anisotropy of the resonance linewidth Δ*B* (Figure 4d) as

(2) $$\Delta \;\;B=\Delta \;\;B_{0}+\alpha \omega /\gamma$$

where Δ*B*_0_ and *αω*/γ are the inhomogeneous and homogenous broadening, respectively, and α is the dimensionless Gilbert damping parameter.^19^

The inhomogeneous linewidth broadening Δ*B*_0_ is attributed to the nonhomogeneous internal magnetic fields arising from the random distribution of the Fe‐islands (Figure 1). The Gilbert damping parameter accounts for the losses of spin angular momentum during the precession of the magnetization around an effective magnetic field ***B*_eff_** that includes the external, internal, and microwave field. When ***B*_eff_** is parallel to the *c*‐axis, the external magnetic field and the magnetization direction are parallel and Δ*B* has a minimum; in contrast, in the out‐of‐plane rotation of ***B*_eff_**, Δ*B* first increases by about 10% for *ϑ*_B_ ≈ 45° and then increases steeply for *ϑ*_B_ approaching a value of *ϑ*_B_ ≈ 90° (Figure 4d). The angular dependence of Δ*B* suggests a magnetic dragging effect due to the noncolllinearity of the external magnetic field and the magnetization direction.^19^ The contribution to the linewidth broadening Δ*B* of the Gilbert parameter (Equation (2)) implies angular momentum losses of the magnetization precession in the magnetic Fe‐islands into the nonmagnetic InSe matrix. This spin‐pump mechanism^5^ occurs at room temperature, offering prospects for the generation of a charge current in InSe via the inverse spin Hall effect.^20^

The uniaxial magnetic anisotropy is observed at room temperature and it depends only weakly on temperature, as assessed by SQUID at low (*T* = 5 K) and room temperature (*T* = 300 K) (Figure S3, Supporting Information). Due to the coexistence of mixed phase states within a system that consists of a diamagnetic InSe crystal and ferromagnetic Fe‐islands (Figure 2), the temperature dependence of the magnetic susceptibility tends to be weak and different from that expected for ferromagnetic γ‐Fe (Figure S3, Supporting Information). Furthermore, the ESR spectra reveal a complex behavior. The weakening of the main ESR lines, *g*_1_ and *g*_2_, with decreasing temperature indicates the emergence of an anisotropic antiferromagnetic (AF) order at a Néel temperature *T*_N_ ≈ 260 K (Figure 5b). An AF order with a Néel temperature *T*_N_ up to 100 K was reported for γ‐Fe thin films with face‐centered cubic (fcc) crystal symmetry obtained by epitaxial growth^21^ or precipitation,^22^ an AF order can emerge when bcc γ‐Fe undergoes a crystal phase transition to fcc below a critical layer thickness^23^ due to stronger magnetic interactions arising from a smaller lattice constant^24^ and/or surface effects^25^; AF and FM orders can also coexist within an Fe‐cluster due to its composite crystal structure.^26^ The coexistence of different magnetic phases in our system may arise from nonequivalent Fe‐atoms in the islands and strain effects at the interface with the diamagnetic InSe, which requires further investigation.

To conclude, recent advances in the science and technology of vdW crystals have demonstrated the potential of this class of materials for novel functional devices. Among these crystals, InSe has emerged as a semiconducting system with unique electronic and optical properties, including high electron mobility^11^ and strong photosensitivity.^12^ Here, we have shown that the formation of crystalline Fe‐islands in InSe induces a uniaxial internal magnetic field (≈1 T) perpendicular to the InSe layers. Thus, this hybrid system, which consists of Fe‐inclusions and a van der Waals crystal, enables the coexistence of magnetic and semiconducting properties within the same structure. Our findings will stimulate further research on magnetism in novel semiconductor materials beyond conventional Si^27^ and GaAs.^28^ Since vdW crystals are compatible with other vdW crystals, magnetic metals, and dielectrics, we envisage further developments and a new class of devices that exploit the magnetic properties of hybrid magnetic‐semiconducting materials. In particular, losses of spin angular momentum during the precession of the magnetization in the ferromagnetic Fe‐islands into the nonmagnetic InSe offer prospects for the generation of a charge current in InSe via the inverse spin Hall effect and its control by the magnetic anisotropy of the crystal. Further developments also include the homogenous incorporation of substitutional Fe‐atoms in InSe, which has recently been proposed as a route to create a homogeneous ferromagnetic semiconductor.^29^

## Experimental Section

*Materials and Structural Studies*: The crystallization of the Fe‐islands occurred along with the formation of the layered InSe vdW crystal during the Bridgman growth. The InSe:Fe melt was contained inside a quartz ampoule and was cooled down slowly using a moving crucible inside the Bridgman furnace.

The crystal structure of all crystals was probed by X‐ray diffraction using a DRON‐3 X‐ray diffractometer that used monochromatic Cu Kα radiation of wavelength λ = 1.5418 Å (Section S1, Supporting Information). Transmission electron microscopy (TEM) experiments were conducted on thin sections of the crystals prepared by focused ion beam scanning electron microscope, FIB‐SEM (FEI Quanta 3D). The crystals were studied using a JEOL 2100F microscope operating at 200 kV, equipped with a Gatan Orius camera and Oxford Instruments X‐Max 80 EDX detector. SEM EDX studies were performed on the crystals using an FEI Quanta 650 operating at 20 kV, equipped with an Oxford Instruments X‐Max 150 detector. The flakes were prepared from the as‐grown crystals by mechanical exfoliation using adhesive tape and then transferred onto a Si/SiO_2_ substrate. Images of the flakes' topography were acquired using an Asylum Research MFP‐3D AFM operated in tapping mode under ambient conditions.

*Magnetic and Optical Studies*: MFM measurements were performed on a Dimension Icon (Bruker) scanning probe microscope (SPM). The MFM probe (Nanosensor PPP MFMR) had a typical spring constant *k* = 2–3 N m^−1^ and curvature radius *r* < 30 nm.^30^ The MFM imaging was carried out using a two‐pass technique. During the first pass, the SPM was operated in the atomic force microscopy mode to determine the topography. During the second pass, the topography line (obtained during the first pass) was retraced while oscillating the probe at a frequency *f* = 69.56 kHz (free‐space amplitude *A*_f_ ≈ 200 nm), maintaining a set distance of *h* = 25 nm between the probe and sample, and recording the cantilever phase change due to probe–sample magnetic interactions. The scan across the sample was conducted at a rate of 0.7 Hz.

ESR measurements were recorded on commercial Bruker EMX and E580 spectrometers operated at Q‐band (34 GHz). The microwave field was perpendicular to the external magnetic field **B** (perpendicular mode). Typical experimental conditions were as follows: modulation amplitude of 0.5 mT, modulation frequency of 50 kHz, and conversion time of 20 ms. The experimental setup for the micro‐PL and Raman studies comprised a He‐Ne laser (λ = 633 nm) or a frequency doubled Nd:YVO_4_laser (λ = 532 nm), an *x–y–z* motorized stage and an optical confocal microscope system equipped with a 0.5 meter long monochromator with a 150 and 1200 g mm^−1^ gratings. The laser beam was focused to a diameter *d* ≈ 1 µm using a 100× objective. Optical experiments were performed at low excitation power (*P* < 0.1 mW) to avoid excessive heating. The signal was detected by a Si charge‐coupled device camera. Magnetometry was performed on a commercial Quantum Design MPMS 3 SQUID.

## Conflict of Interest

The authors declare no conflict of interest.

## Supporting information

Supplementary
