# Supplementary material for: Room Temperature Uniaxial Magnetic Anisotropy Induced By Fe‐Islands in the InSe Semiconductor Van Der Waals Crystal
Source: Adv Sci (Weinh). 2018 May 11;5(7):1800257. doi: 10.1002/advs.201800257 (PMC6051381; doi:10.1002/advs.201800257)
Supplement: Supplementary file 1 — Supplementary [file ADVS-5-1800257-s001.pdf]

Copyright WILEY-VCH Verlag GmbH & Co. KGaA, 69469 Weinheim, Germany, 2018.

Supporting Information

for *Adv. Sci.,* DOI: 10.1002/advs.201800257

Room Temperature Uniaxial Magnetic Anisotropy Induced By Fe-Islands in the InSe Semiconductor Van Der Waals Crystal

Fabrizio Moro, Mahabub A. Bhuiyan, Zakhar R. Kudrynskyi, Robert Puttock, Olga Kazakova, Oleg Makarovsky, Michael W. Fay, Christopher Parmenter, Zakhar D. Kovalyuk, Alistar J. Fielding, Michal Kern, Jorisvan Slageren, and Amalia Patanè**

DOI: 10.1002/(advs.201800257)
Article type: Communication

Supplementary information

Room temperature uniaxial magnetic anisotropy induced by Fe-islands in the InSe semiconductor van der Waals crystal

Fabrizio Moro, Mahabub A. Bhuiyan, Zakhar R. Kudrynskyi, Robert Puttock, Olga Kazakova, Oleg Makarovsky, Michael W. Fay, Christopher Parmenter, Zakhar D. Kovalyuk, Alistar J. Fielding, Michal Kern, Joris van Slageren, and Amalia Patanè**

Dr. F. Moro, M. A. Bhuiyan, Dr. Z. R. Kudrynskyi, Dr. O. Makarovsky, Prof. A. Patanè
School of Physics and Astronomy, The University of Nottingham, NG7 2RD, Nottingham, United Kingdom
E-mail: amalia.patane@nottingham.ac.uk

Dr. F. Moro
Department of Physics, Chemistry and Biology, Linköping University, 581 83 Linköping, Sweden
E-mail: fabrizio.moro@liu.se

R. Puttock, Dr. O. Kazakova
National Physical Laboratory, Hampton Road, TW11 0LW, Teddington, United Kingdom

Dr. M. W. Fay, Dr. C. Parmenter
Nanoscale and Microscale Research Centre, The University of Nottingham, NG7 2RD, Nottingham, United Kingdom

Prof. Z.D. Kovalyuk
Institute for Problems of Materials Science, The National Academy of Sciences of Ukraine, 58001, Chernivtsi, Ukraine

Dr. A. J. Fielding
School of Chemistry and Photon Science Institute, The University of Manchester, Oxford Road, M13 9PL, Manchester, United Kingdom.
School of Pharmacy and Biomolecular Sciences, Byrom Street, L3 3AF, Liverpool, United Kingdom.

M. Kern, Prof. J. van Slageren,
Institut für Physikalische Chemie, Universität Stuttgart, Pfaffenwaldring 55, D-70569, Stuttgart, Germany

S1. X-ray diffraction

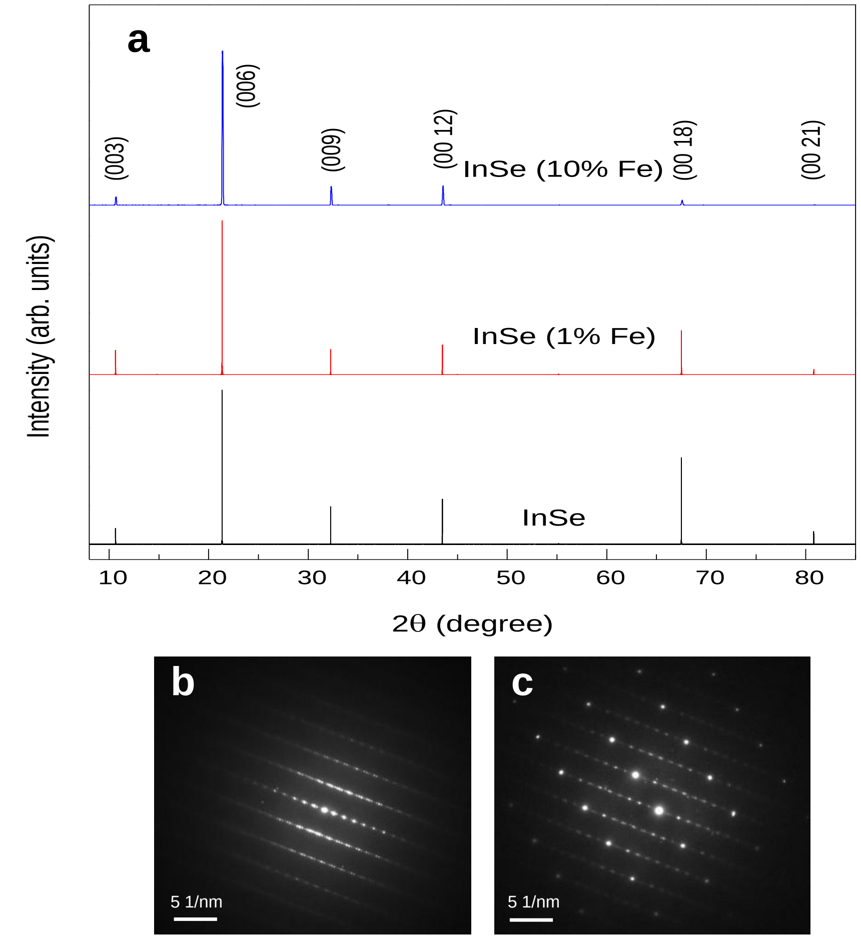

Figure S1: a) X-ray diffraction patterns for InSe with nominal Fe-content of 0%, 1% and 10%. b-c) Electron diffraction patterns of InSe without (b) and with (c) Fe.

**Fe (%)**	$a = b$ **(Å)**	c **(Å)**
0	4.002 ± 0.003	24.950 ± 0.014
1	4.012 ± 0.008	24.953 ± 0.002
10	4.011 ± 0.017	24.905 ± 0.032

Table S1: Lattice parameters (a , b and c) from XRD studies of InSe:Fe crystals.

S2. Raman spectroscopy

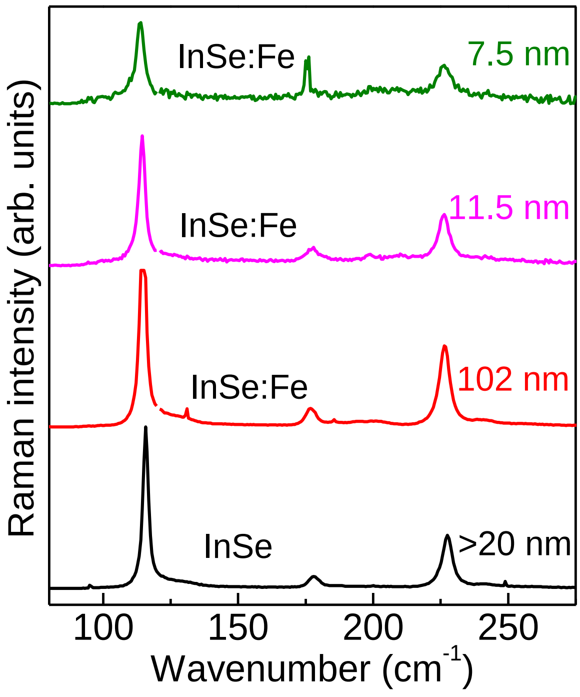

Figure S2: Normalized Raman spectra for different layer thickness of exfoliated flakes from InSe and InSe with nominal Fe content of 10%.

S3. SQUID measurements

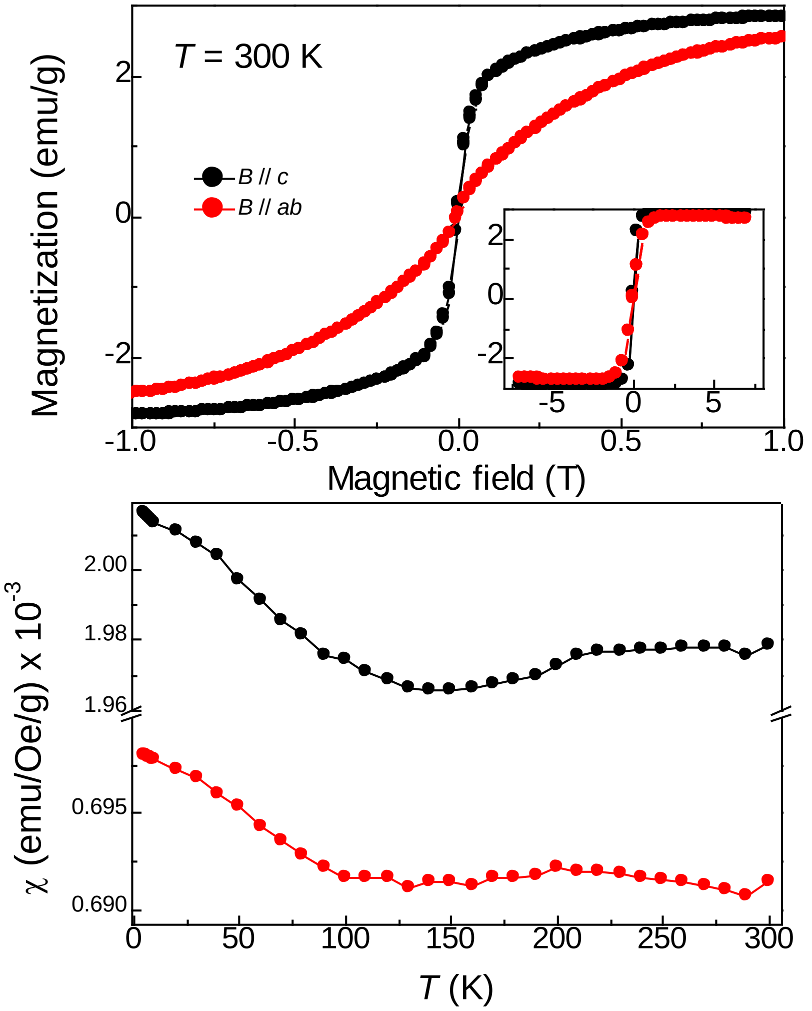

Figure S3: Magnetic field dependent magnetization at T = 300 K (top) and temperature dependence of the magnetic susceptibility (bottom) for an InSe crystal with nominal Fe = 10% oriented parallel to the *c*-axis and to the *ab*-plane.

S4. ESR line g_3

Temperature dependent ESR spectra for B parallel to the c-axis

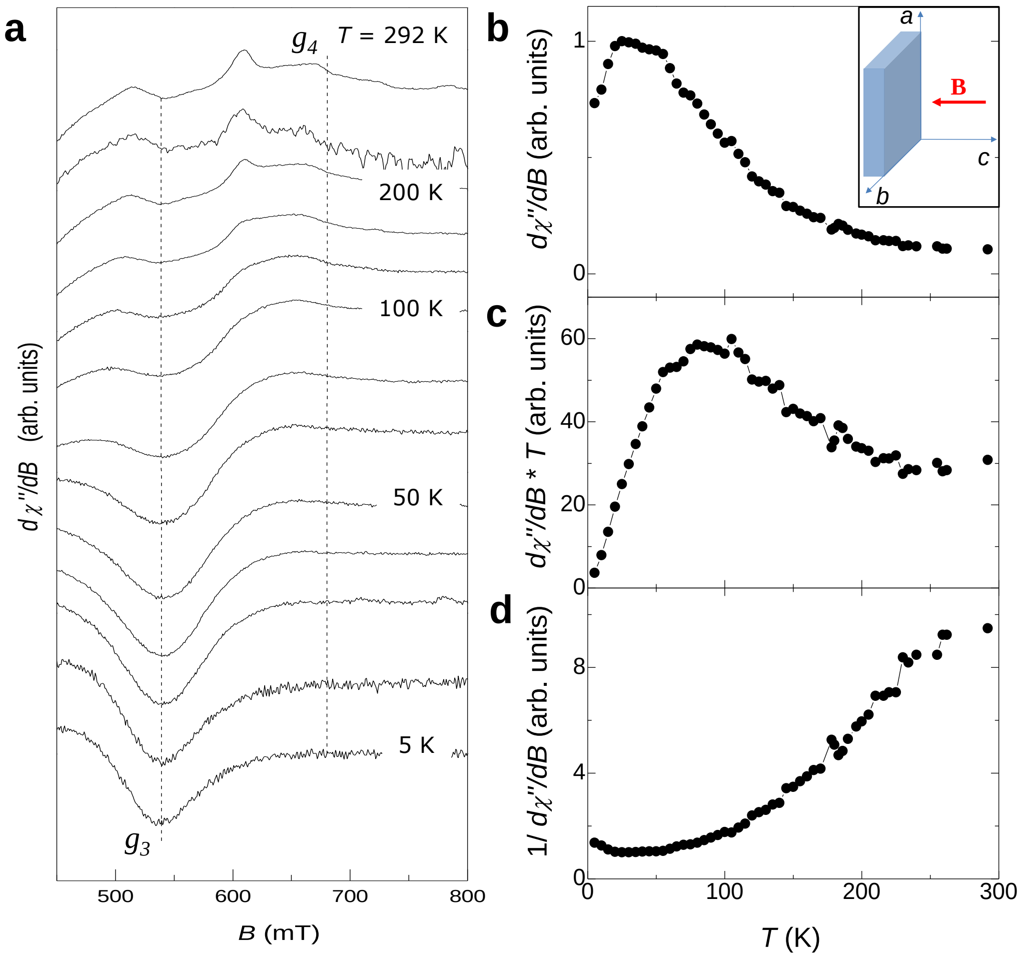

Figure S4: a) Temperature-dependent ESR spectra for an InSe crystal with nominal Fe = 10% (Q-band and **B** parallel to the c-axis, see inset). The ESR spectra are shifted along the vertical axis for clarity. Temperature values from top to bottom are T = 292 K, 250 K, 200 K, 150 K, 120 K, 100 K, 80 K, 60 K, 50 K, 40 K, 30 K, 10 K and 5 K. b-c-d) ESR intensity, ESR intensity times temperature and reciprocal ESR intensity of resonance g_3 versus T.

S5. ESR line g_4

Temperature dependent) ESR spectra for B parallel to the ab plane

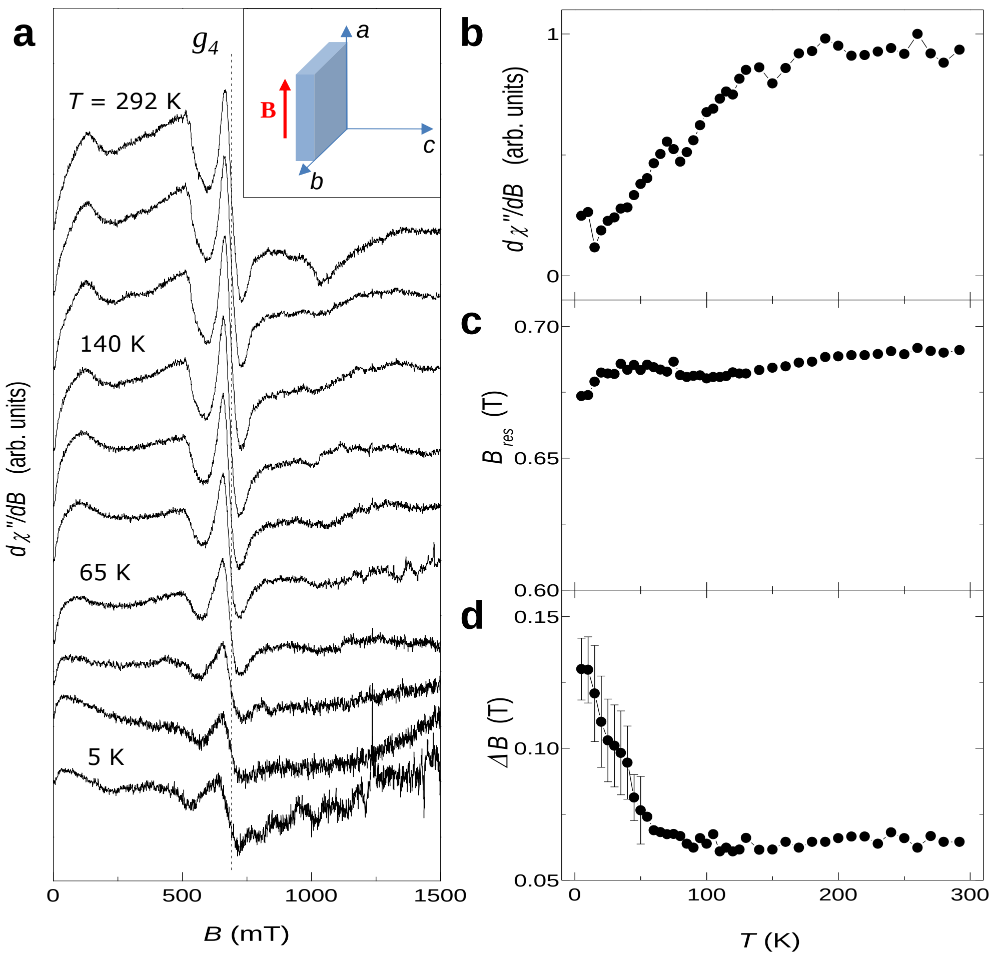

Figure S5: a) Temperature-dependent ESR spectra for InSe with nominal Fe = 10% (*Q*-band and **B** in the *ab*-plane, see inset). The ESR spectra are shifted along the vertical axis for clarity. Temperature values from top to bottom are *T* = 292 K, 220 K, 200 K, 140 K, 115 K, 95 K, 65 K, 45 K, 35 K and 5 K. b-c-d) ESR intensity, resonance field and linewidth of resonance g_4 versus *T*.

S6. ESR line g_4

Angular rotation dependent ESR spectra for B parallel to the ab plane

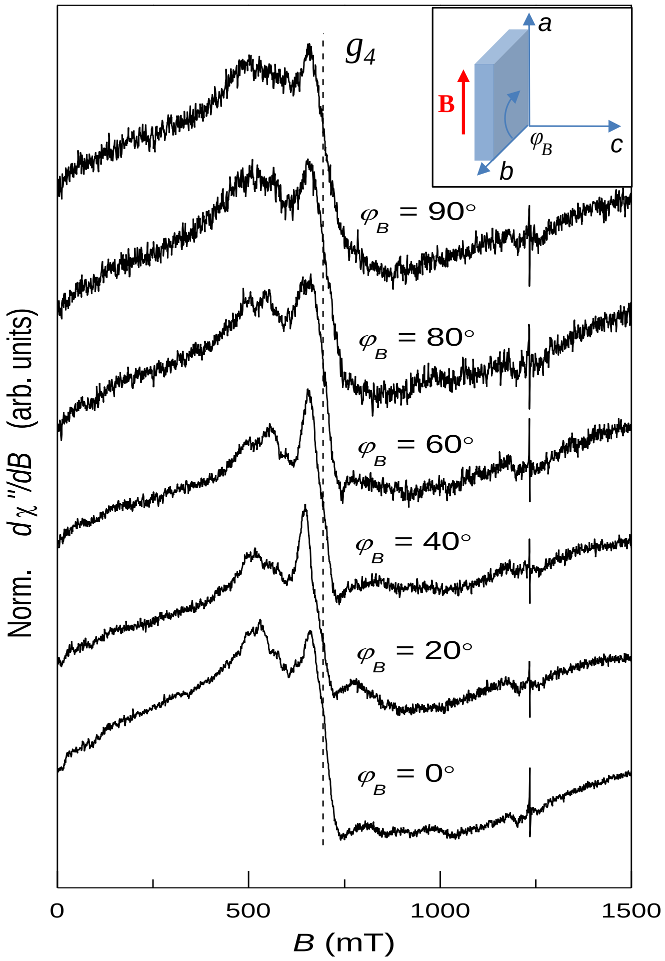

Figure S6: Angular rotation (**B** in the *ab*-plane, see inset) dependent ESR spectra of InSe with nominal Fe = 10% (Q-band and T = 292 K). The ESR spectra are shifted along the vertical axis for clarity.
